# Differences in Tolerance to Host Cactus Alkaloids in *Drosophila koepferae* and *D. buzzatii*

**DOI:** 10.1371/journal.pone.0088370

**Published:** 2014-02-10

**Authors:** Ignacio M. Soto, Valeria P. Carreira, Cristian Corio, Julián Padró, Eduardo M. Soto, Esteban Hasson

**Affiliations:** 1 Instituto de Ecología, Genética y Evolución de Buenos Aires, Consejo Nacional de Investigaciones Científicas y Tecnológicas, Buenos Aires, Argentina; 2 Departamento de Ecología, Genética y Evolución, Facultad de Ciencias Exactas y Naturales, Universidad de Buenos Aires, Buenos Aires, Argentina; AgroParisTech, France

## Abstract

The evolution of cactophily in the genus *Drosophila* was a major ecological transition involving over a hundred species in the Americas that acquired the capacity to cope with a variety of toxic metabolites evolved as feeding deterrents in Cactaceae. *D. buzzatii and D. koepferae* are sibling cactophilic species in the *D. repleta* group. The former is mainly associated with the relatively toxic-free habitat offered by prickly pears (*Opuntia sulphurea*) and the latter has evolved the ability to use columnar cacti of the genera *Trichocereus* and *Cereus* that contain an array of alkaloid secondary compounds. We assessed the effects of cactus alkaloids on fitness-related traits and evaluated the ability of *D. buzzatii and D. koepferae* to exploit an artificial novel toxic host. Larvae of both species were raised in laboratory culture media to which we added increasing doses of an alkaloid fraction extracted from the columnar cactus *T. terschekii*. In addition, we evaluated performance on an artificial novel host by rearing larvae in a seminatural medium that combined the nutritional quality of *O. sulphurea* plus amounts of alkaloids found in fresh *T. terschekii*. Performance scores in each rearing treatment were calculated using an index that took into account viability, developmental time, and adult body size. Only *D. buzzatii* suffered the effects of increasing doses of alkaloids and the artificial host impaired viability in *D. koepferae*, but did not affect performance in *D. buzzatii*. These results provide the first direct evidence that alkaloids are key determinants of host plant use in these species. However, the results regarding the artificial novel host suggest that the effects of alkaloids on performance are not straightforward as *D. koepferae* was heavily affected. We discuss these results in the light of patterns of host plan evolution in the *Drosophila repleta* group.

## Introduction

Phytophagous insects are excellent model systems to investigate the genetic and ecological basis of adaptation and interspecific divergence, since their host plants constitute the most immediate environmental factor affecting early life cycle stages [1]. In this regard, the role of ecology in speciation has been systematically evaluated [2], [3], [4] and a recent metanalysis involving groups of angiosperms, fishes, frogs, birds, pigeons, butterflies and fruit flies revealed a link between ecological divergence and reproductive isolation [5], [6]. Changes in habitat/diet were shown to be positively associated with reproductive isolation in insects [5], [7] supporting the notion that ecological consequences of host plant shifts may be responsible for the remarkable diversity of phytophagous groups.

Host specificity in phytophagous insects especially in monophagous species, is thought to be based on chemical and/or nutritional characteristics of the plant [1], [8]. Thus, shifts to new host plants often involve challenges to exploit a new food source, face chemically diverse environments (including potentially toxic compounds), new mating environments, parasitoids, bacteria and fungi [9], [10], [11]. Hence, host plant shifts may accelerate divergence in features associated with performance in new hosts [12], [13], [14], [15], [16], and sensory systems, like those involved in smell and taste [17], [18]. Similarly, changes in morphology associated with host plant shifts are well documented in insects [17], [19], [20], [21]. However, many other factors may influence host choice and the evolution of specialists versus generalists. For instance, chemical properties along with other features such as temporal and spatial availability are relevant factors that influence the suitability of hosts. Studies of host plant chemistry have resulted in a general understanding of insect-plant relationships (reviewed in [1]). Some host plants have evolved metabolic pathways responsible for an extraordinary variety of secondary metabolites that reduce damage by herbivores [1]. As a matter of fact, the chemical particularities of plants are thought as relevant factors that shape the ensemble of insects that use a plant as feeding or breeding site [8].

Most species in the genus *Drosophila* are saprophytophagous and breed on necrotic plant tissues and feed upon the microorganisms associated to the decaying process [22]. The ecology of *Drosophila* breeding sites has been an issue of interest for evolutionary biologists because of the prominent role that several members of the genus played in genetics and evolution [23]. Among such groups the *repleta* group (subgenus *Drosophila*) radiated in the New World due to the ability of flies to utilize decaying cacti as breeding substrates [24]. This capacity allowed some species subgroups to invade and diversify in the deserts of the Americas, areas that are rather inhabitable for other *Drosophila* [24], [25].

The radiation of the Cactaceae has been accompanied by the acquisition of a broad battery of secondary metabolites (allelochemicals) as alkaloids, medium chain fatty acids, sterol-diols, and triterpene glycosides. The latter serve as feeding deterrents and isoquinoline alkaloids obstruct neurotransmission (reviewed in [26]). Moreover, it has been argued that toxic compounds affect fitness related traits [28], [29] and determine patterns of host plant use in cactophilic *Drosophila* that inhabit the Sonoran Desert [27]. Moreover, in some cases the effects are so dramatic that “mistakes” in the choice of a breeding substrate might result in the death of the insect. As an example, *D. pachea* is restricted to senita cactus (*Lophocereus schotii*) due to a strict nutritional requirement for Δ^7^ sterols (only found in senita) to complete development (reviewed in [26], [30], [31]).

The *D. buzzatii* cluster is an ensemble of at least seven species in different stages of divergence and varying degrees of host specialisation [32]. The sibling species *D. buzzatii* and *D. koepferae* are sympatric in most of the distribution range of the latter in the areas arid lands of northwestern Argentina and southern Bolivia [33], [34]. The former breeds primarily on the decaying cladodes of several species of the genus *Opuntia* (prickly pears) and secondarily on columnar cacti of the genera *Cereus* and *Trichocereus*, whereas the reverse is true for *D. koepferae* [35]. Since divergence, *D. buzzatii* and *D. koepferae* have evolved differences in life-history and morphological traits expressed in both primary and secondary natural hosts [36], [37], [38], [39], and also a remarkable oviposition preference on their respective primary natural hosts [15], [40]. These observations have been interpreted as adaptations that allow flies to efficiently exploit resources that differ markedly in spatial and temporal predictability as well as in chemical properties [34], [35], [36], [41]. *Opuntia* species contain acids that are typical of succulents (*eucomic, phorbic* and *piscidic*), lipids, terpenes, free sugars and phenolic compounds [42] while cacti in the genus *Trichocereus* contains alkaloids such as *candicine* and *trichocereine* in *T. candicans* and *T. terschekii* respectively [43]. These chemical particularities have led to the suggestion that alternative cactus hosts may represent different chemical environments for the larvae that develop in the decaying plant tissues and for adult flies that feed on them [35], [36], [44]. In a previous study, we showed that alkaloids extracted from *T. terschekii* decrease viability and adult body size in *D. buzzatii* [45]. However, we did not test the hypotheses that alkaloids exerted a differential effect on performance in the columnar dweller *D. koepferae* vs. *D. buzzatii*.

Here, we carry out the first comparative assessment of the effect of cactus alkaloids on life history traits and fitness in *D. buzzatii* and *D. koepferae* and the evaluation of the intrinsic capabilities of each species to exploit a novel toxic host.

## Materials and Methods

### Collection of flies, cacti and stocks maintenance

Fly stocks used in this study derived from flies collected in San Agustín del Valle Fértil (30° 31′13′ ´ S, 67° 34′05′ ´ W; Province of San Juan, Argentina), where *D. buzzatii* and *D. koepferae* coexist [41]. Flies were collected by net sweeping over fermented banana baits. Collection permits for both flies and cacti tissues were issued to IMS and JP by the Conservation Management and Protected Areas (Secretariat of Environment and Sustainable Development. Province of San Juan, File N° 1300-0236-13).

In the laboratory, wild flies were separated by sex and females were allowed to oviposit in vials in order to establish isofemale lines. As females of both species are morphologically indistinguishable isofemale lines were identified to species by inspecting genitalia of F1 males [46]. The offspring of 30 isofemale lines were mixed in equal numbers to establish two outbred stocks, one for each species, that were maintained in standard laboratory instant medium (Carolina Supplies) under a 12:12h light/dark photoperiod at 25±1°C.

Fresh tissues and fermenting cladodes of *O. sulphurea* and stems of *T. terschekii* were also returned to the laboratory from the same locality [40], [41]. Pieces of fresh cacti were stored at −20°C and cactus necroses of each species were maintained at 4° C in 25×25×15 plastic containers with sterilized cotton caps where fresh cactus was added every month (during the three months of the experiment).

### Extraction and isolation of alkaloids from *T. terschekii*

Fresh tissues of *T. terschekii* were ground and blended with EtOH (1 l/ 1 kg tissue) and then filtered. The organic extract was concentrated on a rotatory evaporator to an aqueous suspension and acidified with 500 ml of 10 % HCl. The aqueous acidic fraction was partitioned between CH_2_Cl_2_ (extracted three times with 500 mL) and water to yield a dichloromethane fraction and a water soluble fraction (see [45]). The former was evaporated in a rotatory evaporator yielding a non-basic fraction containing acid lipid soluble compounds (e.g. terpenoids, fatty acids, sterols, aromatic and other compounds). This fraction, hereafter referred to as the “non-alkaloid fraction; NA”, was included as a separate treatment in the experiments described below to investigate its possible biological effects, since, *a priori*, we did not know which fraction contained potential toxic compounds other than alkaloids responsible for the differential effects that *T. terschekii* has on *D. buzzatii* and *D. koepferae*. The organic fraction was dried to yield a crude alkaloid fraction, hereafter referred to as the “Alkaloid fraction; A”. The identification of the active components in the alkaloid fraction was accomplished via mass spectrometry. We confirmed the presence of three compounds: trichocereine, N-dimethylmescaline, a phenylethylamine alkaloid typical of this species, mescaline and the analogue α-methylmescaline [45]. The natural concentration of alkaloids in fresh *T. terschekii* estimated from the collected material was 0.33 mg/g of wet fresh weight and 4.50 mg/g in the dry sample (0.3% w/w). Both the alkaloid (A) and the non-alkaloid (NA) fraction were solubilized in dimethyl sulfoxide (DMSO 100 µgr/ml) and used to design the artificial diets used in the bioassays. The same extraction protocol was applied to *O. sulphurea* samples but no traces of alkaloids were detected.

### Experimental design

In order to obtain first-instar larvae, two egg-collecting chambers for each species were set up with a Petri dish containing egg laying medium (2% agar + commercial yeast). One hundred pairs of sexually mature flies of the same stock were released into each chamber. Petri dishes were removed 12 h later, inspected for the presence of eggs and incubated for another 24 hours to allow larval hatching. For each treatment, groups of 30 first instar larvae were randomly sampled from the plates and seeded in vials with the corresponding rearing medium (five vials or replicates per treatment). All vials contained 0.8 g of standard *Drosophila* instant medium (Carolina Biological Supply Company, Burlington, NC) hydrated with 4 mL of a solution containing sodium methylparaben (Nipagin, 0.02 g/v) as fungicide and the corresponding amount of the alkaloid or non-alkaloid fraction depending on the experiment (see below).

For the assessment of the effects of alkaloids on each species' performance, we raised batches of larvae in vials containing laboratory culture medium and increasing doses of the alkaloid fraction (A-treatments). The first set of vials contained standard laboratory medium plus the alkaloid fraction to a final alkaloid concentration that was close to its concentration in cactus tissues (A1X treatment). The other two sets of vials contained standard lab medium and alkaloid concentrations that were 50% (A1.5X) and 100% (A2X) higher than in the A1X treatment. The rationale of including treatments varying in the concentration of the alkaloid fraction was to uncover natural variation that flies may encounter in nature. Actually, alkaloid concentration may vary depending on cactus age and other ecological variables as soil properties and elevation [47], [48], [49]. In addition, water evaporation may contribute to increase alkaloids concentration in the rotting pocket during the decaying process [50].

We also evaluated the possible effect of the non-alkaloid fraction obtained during the extraction and isolation of alkaloids because some columnar cacti contain other secondary compounds such as triterpene glycosides, sterol diols and rare fatty acids [27] that may affect flies. For instance, cis-vaccenic acid, a rare isomer of oleic acid is abundant in *T. terschekii* [51] and previously reported as a pheromone precursor in insects [52] [53].

Thus, the non-alkaloid fraction might also account, apart from alkaloids, for the differential effects that rotting cacti have on performance. In these experiments, we prepared three sets of vials with increasing doses of the non-alkaloid fraction. One set contain the same concentration of the NA fraction measured in fresh tissues (NA1X treatment) and the other two contained 1.5 (NA1.5X) and 2 (NA2X) times the amount added to the first set.

Finally, we evaluated if differences in performance between flies raised in media prepared with *T. terschekii* and *O. sulphurea* were due solely to the presence of alkaloids or, alternatively, to an interaction with the overall quality of the plant tissue. To test this hypothesis, we created an artificial host prepared with fermented tissues of *O. sulphurea* plus an amount of the alkaloid fraction that matched the alkaloid concentration in fresh tissues of *T. terschekii*, an artificial “novel host”. Then, we raised batches of 30 larvae of *D. buzzatii* and *D. koepferae* in this artificially created diet and, as controls, in vials containing semi-natural media prepared with *T. terschekii* or *O. sulphurea* rotting tissues.

### Traits scored

Performance in each rearing condition was measured by means of the study of larval to adult viability (LV), developmental time (DT) and wing size (WS) as a proxy for body size. LV was estimated as the proportion of emerged adults relative to the number of larvae seeded in each vial/replicate of each treatment. DT was estimated as the elapsed time in hours from the time of transfer of first instar larvae to vials until adult emergence. For the measurement of this trait, emerged flies were collected and sexed every four hours. The right wings of adult males were removed and mounted on slides and images of wings were obtained with a digital camera mounted on a microscope. Ten landmarks were digitized using TpsDig [54] at the intersection of veins or at the intersection of veins with the margins of the wing following Soto et al. [39]. As a measure of WS, we calculated the centroid size of each individual configuration of landmarks, using the square root of the sum of the squared distances of each landmark to the centroid of the configuration, [55].

### Statistical analyses

We combined LV, DT and WS into a single index that gives a proxy of overall host performance. We calculated a relative performance index (RPI; modified from [56], [57] for each vial using: 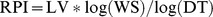



This equation is very straightforward since LV and WS are directly related and DT is inversely related to the efficiency in the use of a rearing substrate [58]. RPI Index combines the effect of viability, development time and size. However, development time and size could themselves be correlated in *Drosophila* (with opposite effects on the inclusive fitness) [59] but also see [60]. Thus, these two parameters may not be independent and inclusion of both in the calculation of RPI may provide a biased estimate of performance. Therefore we evaluated the degree of independence in our data through the estimation of Pearson correlations among traits for each species in every experimental condition.

Responses to increasing concentrations of alkaloid and non-alkaloid fractions were evaluated by means of regression analyses of performance on the dose in A and NA treatments for each species. In the regressions the value of the performance index calculated for each replicate was the dependent variable. Additionally we performed a test of Homogeneity of Slopes in order to evaluate differences in response to alkaloid and non-alkaloid treatments within each species. In these analyses, the value of the performance index and the concentration of the respective fraction were considered as the dependent and independent variables, respectively, and fraction, alkaloid vs. non-alkaloid, was included as a categorical independent factor.

Performance variation among flies raised in media prepared with *T. terschekii*, *O. sulphurea* and *O. sulphurea* plus alkaloids was evaluated by means of a two-way ANOVA with species and host as independent fixed variables. We also calculated coefficients of variation (CV) for each treatment as the ratio between the standard deviation and mean performance using the means calculated for each replicate as input data. As this coefficient measures the dispersion of data points (i.e. means of replicates) around the mean value corresponding to each treatment, it was used to compare the degree of variation among treatments even if their means were different. As there is no variance for each CV, confidence intervals were constructed using bootstrap estimates of the coefficient [61].

We also explored whether there was a correlation between performance and CV in each species by calculating Pearson product moment correlations. All data were inspected for normality and RPI was not normally distributed with a moderately positive skewness. Thus, in order to fulfill normality and homoscedasticity assumptions, we applied the square root transformation to the data (following [62]) before analyses. All statistical analyses were performed using GLM implemented in the STATISTICA 6.0 software package [63] except for bootstrapping that was performed using PoopTols [64].

## Results

**Figure 1:**
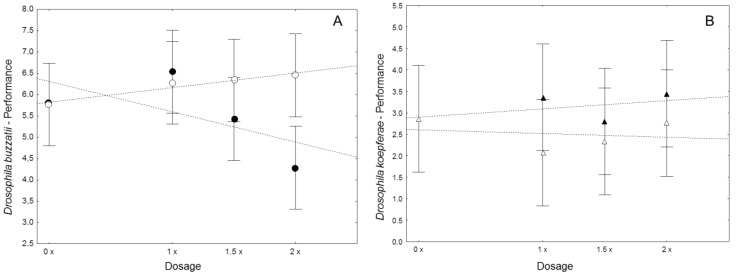
Performance as a function of concentration. Mean relative performance (and 95 % confidence intervals) as a function of concentration for a) *D. buzzatii* and b) *D. koepferae* reared in medium with the alkaloid fraction (black symbols) or the non-alkaloid fraction (white symbols) extracted from the columnar cactus *T. terschekii*. Linear trends are shown.

Mean values for all traits measured in each experiment and treatment as well as the respective performance score and CV are reported in Table 1. Only *D. buzzatii* was significantly affected by the presence of alkaloids in the rearing medium. The regression of performance on alkaloid dose was significant in *D. buzzatii* but not in *D. koepferae* (Table 2, Figure 1). Increasing doses of the alkaloid fraction affected performance in *D. buzzatii* by decreasing LV and extending DT, but did not affect wing size (Table 2). However, alkaloids concentration did not affect any of the life history traits in *D. koepferae* (Table 1 and Table 2). Both species failed to show any response to the presence of the non-alkaloid fraction (Table 2). Though a trend of increasing viability at higher concentrations of the non-alkaloid fraction was observed in both species in Figure 1, regressions were not significant for *D. buzzatii* (p  =  0.46) or *D. koepferae* (p  =  0.41) (Figure 1). Heterogeneity of the regressions slopes of performance for alkaloid and non-alkaloid concentrations were significant in *D. buzzatii* (*F_1,36_*  =  4.77, p  =  0.035) but not in *D. koepferae* (*F_1,36_*  =  0.24, p  =  0.623). Regarding possible correlations among traits conforming the RPI index, a significant negative correlation was detected between viability and developmental time only in the alkaloid-increasing treatments in both species (*r*  =  −0.58 and *r* =  −0.53 for *D. buzzatii* and *D. koepferae* respectively). However, development time and size, regardless the expectation, did not show a significant correlation in any treatment for any species supporting it inclusion in the RPI index as different fitness proxies.

**Table 1:** Performance scores and assessed traits.

	*D. buzzatii*					*D. koepferae*				
Treatments	Viability (Proportion)	Developmental time (hours)	Wing Size (log N°pixels)	Performance	Performance CV	Viability (Proportion)	Developmental time (hours)	Wing Size (log N°pixels)	Performance	Performance CV
Control	0.83 (0.05)	251.62 (5.12)	38.528 (0.003)	5.77 (0.35)	0.206	0.41 (0.08)	323.99 (6.46)	39.904 (0.004)	2.86 (0.55)	0.642
A1	0.93 (0.05)	235.72 (1.43)	38.517 (0.009)	6.53 (0.37)	0.125	0.49 (0.10)	326.33 (5.29)	39.879 (0.004)	3.36 (0.71)	0.472
A2	0.78 (0.04)	255.63 (8.15)	38.516 (0.014)	5.42 (0.29)	0.120	0.41 (0.09)	332.49 (3.3)	39.876 (0.007)	2.79 (0.63)	0.505
A3	0.62 (0.13)	270.95 (13.44)	38.516 (0.019)	4.28 (0.92)	0.478	0.50 (0.04)	328.41 (2.39)	39.863 (0.004)	3.44 (0.30)	0.196
NA1	0.89 (0.04)	241.43 (7.11)	38.524 (0.019)	6.28 (0.28)	0.099	0.30 (0.08)	324.54 (3.14)	39.889 (0.011)	2.07 (0.57)	0.612
NA2	0.91 (0.04)	248.03 (4.77)	38.509 (0.017)	6.33 (0.26)	0.091	0.34 (0.08)	330.30 (4.92)	39.878 (0.005)	2.33 (0.52)	0.498
NA3	0.93 (0.04)	252.92 (4.75)	38.520 (0.009)	6.45 (0.25)	0.086	0.40 (0.03)	326.19 (2.71)	39.887 (0.005)	2.76 (0.19)	0.156
*O. sulphurea*	0.82 (0.05)	207.81 (21.43)	38.521 (0.002)	5.83 (0.35)	0.149	0.74 (0.04)	266.64 (0.87)	39.915 (0.005)	5.32 (0.27)	0.123
*T. terschekii*	0.74 (0.05)	239.26 (4.08)	38.523 (0,002)	5.20 (0.35)	0.166	0.71 (0.04)	266.63 (0.38)	39.901 (0.005)	5.03 (0.26)	0.129
Artificial Host a	0.73 (0.05)	249.29 (4.08)	38.516 (0.006)	5.08 (0.38)	0.184	0.17 (0.02)	321.45 (10.07)	33.193 (6.638)	0.997 (0.23)	0.577

Mean values for each trait measured (and standard error) in each treatment and the corresponding performance index and coefficient of variation for both *D. buzzatii* and *D. koepferae.*

a *O. sulphurea* + alkaloids from *T. terschekii*.

**Table 2:** Regression slopes of assessed traits.

	*D. buzzatii*				*D. koepferae*			
Increasing fraction	Viability	Developmental time	Wing Size	Performance	Viability	Developmental time	Wing Size	Performance
Alkaloids	−0.59*	0.63*	−0.08	−0.47*	−0.04	0.11	−0.54*	−0.07
Non-alkaloids	0.18	0.39	−0.04	0.18	−0.03	0.08	−0.003	−0.2

Slope values of regressions for each trait in both increasing fraction treatments (Alkaloids and Non- alkaloids concentrations) for both *D. buzzatii* and *D. koepferae.* Asterisks denote significant regressions (p<0.05).

### Performance in cactus media

**Figure 2:**
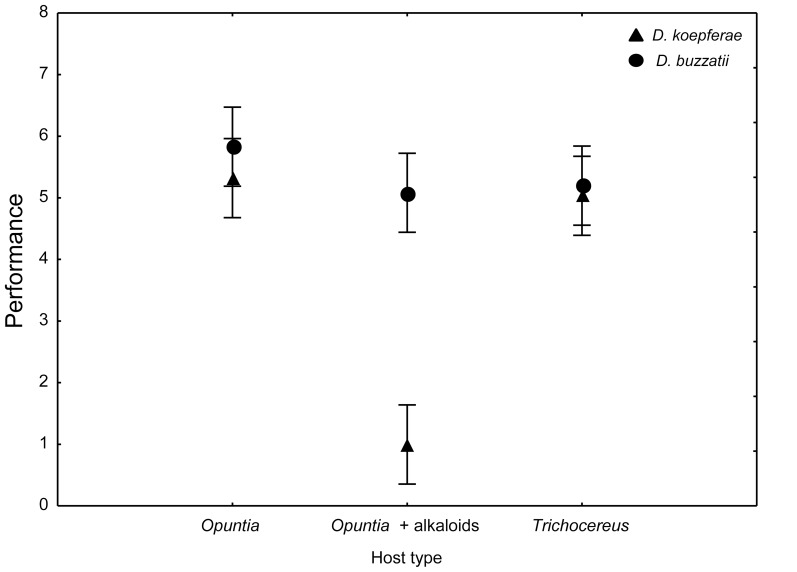
Performance in host cacti. Mean relative performance (and 95 % confidence intervals) for *D. buzzatii* (circles) and *D. koepferae* (triangles) reared in their natural hosts (*O. sulphurea* and *T. terschekii*) and an artificial novel host made with *Opuntia* tissue added with alkaloids extracted from the columnar *T. terschekii*.

Analysis of variance of host dependent performance revealed significant differences between species, where *D. buzzatii* showed, on average, greater scores than its sibling (*F_1, 30_* =  38.24, p< 0.01) between hosts (*F_2, 30_* =  36.93, p< 0.01) and a significant host x species interaction (*F_2, 30_* =  23.83, p< 0.01). Both species showed comparable performances in both natural hosts, but radically different responses when raised on the novel host. Performance differences between *D. buzzatii* reared in the *Opuntia* + alkaloid medium and *T. terschekii* medium were not significant. To the contrary, *D. koepferae* reared in the novel host exhibited significantly reduced performance as compared to flies raised in *O. sulphurea* and *T. terschekii* (Figure 2).

### Coefficients of variation

**Figure 3:**
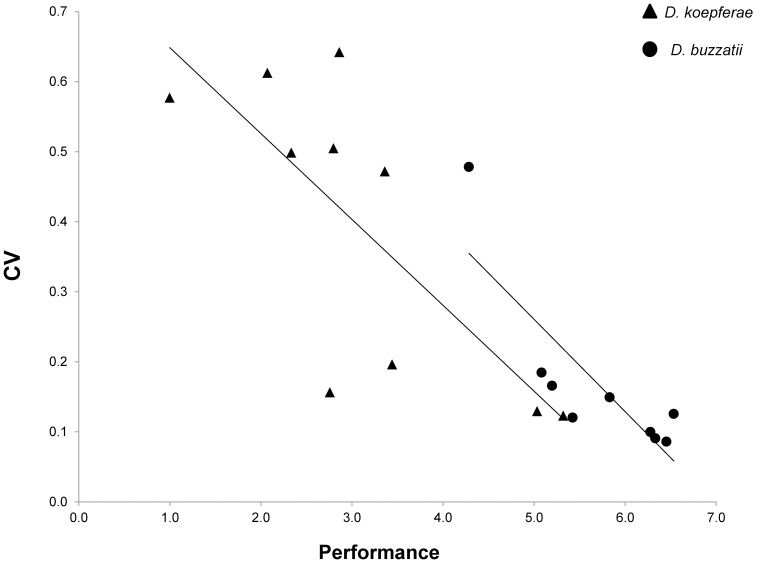
Coefficient of Variation and Performance correlation. Association between the coefficient of Variation (CV) and the mean relative performance considering all treatments in *D. buzzatii* (circles) and *D. koepferae* (triangles). Linear trends are shown.

**Figure 4:**
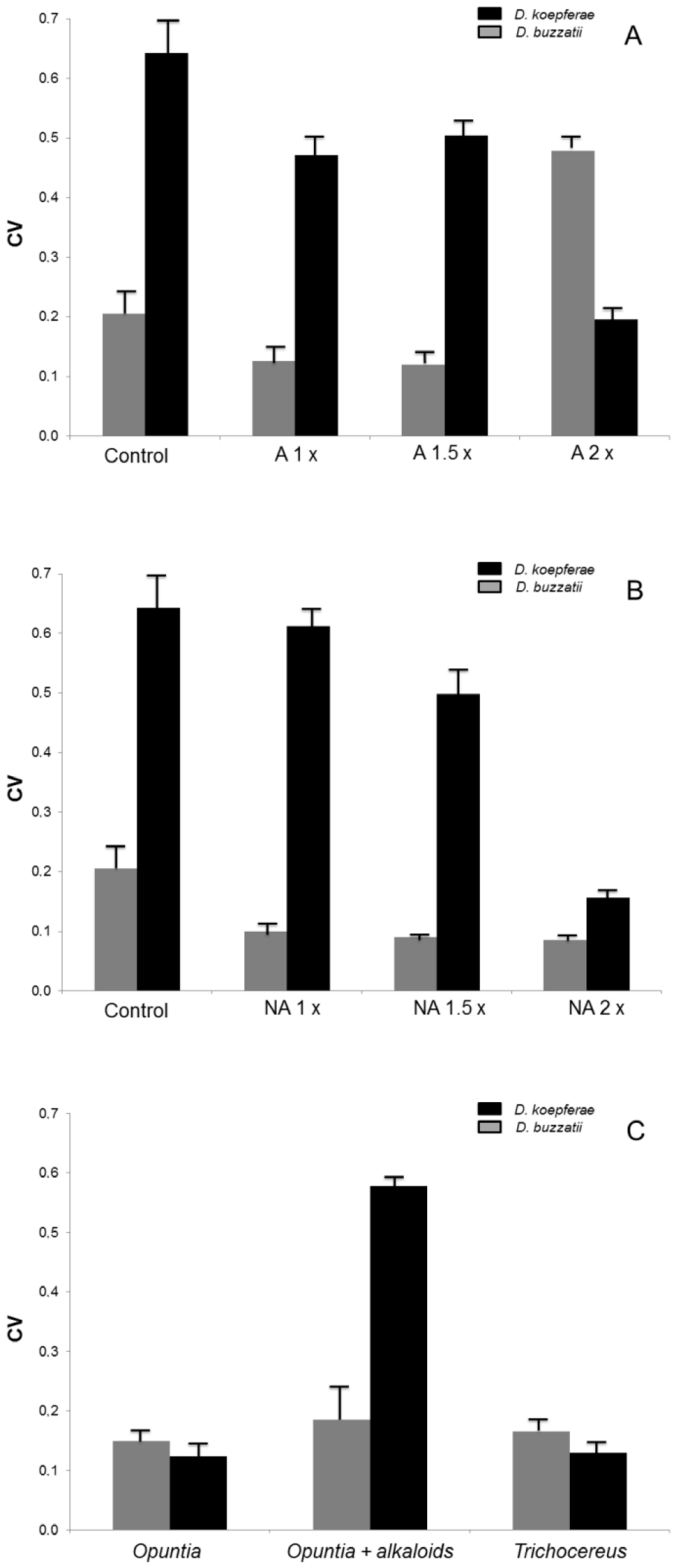
Treatments Coefficients of Variance. Coefficients of variance (CVs) for a) increasing concentrations of the alkaloid fraction extracted from *T. terschekii*, b) increasing concentrations of the non-alkaloid fraction extracted from *T. terschekii* and c) the natural hosts and the novel artificial host for *D. buzzatii* (grey bars) and *D. koepferae* (black bars). Confidence intervals were estimated via bootstrap (see text).

There was a significant negative correlation between mean performance and CV in both species (r  =  −0.83 for *D. buzzatii* and r  =  −0.75 in *D. koepferae*, both p< 0.05; Figure 3) when all treatments were considered jointly. Hence, treatments in which flies had inferior performance also displayed greater variance among replicates (Figure 4). For alkaloid treatments, heterogeneity among samples increased with concentration in *D. buzzatii* but decreased in *D. koepferae* (Figure 4a). For treatments with the non-alkaloid fraction, higher concentrations were associated with lower among sample variance in both species (although this was more evident in *D. koepferae*; Figure 4b). The analysis of performance in the two types of cactus hosts showed that, along with the reduced performance of *D. koepferae* in the *O. sulphurea* + alkaloids medium, we detected a pronounced and concomitant increment of the CV (Figure 4c).

## Discussion

Dose-dependent effects of alkaloid fractions extracted from fresh tissues of *T. terschekii* had detrimental effects on performance in *D. buzzatii* but not in its sibling *D. koepferae.* The alkaloid fraction added to the rearing medium decreased viability and extended developmental time but did not affect wing size. However, *D. koepferae* did not show any significant response to increasing concentrations of alkaloids extracted from its natural host in any of these fitness components suggesting a well-developed tolerance or some similar kind of specialization. Egg-to-adult viability and mean development time are fitness parameters sensitive to environmental conditions and are known to be affected by alkaloids in several species of desert *Drosophila* [65], [66].

Conversely, increasing concentrations of the non-alkaloid fraction did not affect performance in *D. buzzatii* or in *D. koepferae* thus limiting the detrimental effects of the rearing cacti to the alkaloids-enriched portion. The analyses of the coefficients of variation of different treatments showed the other side of the same phenomenon. CVs were greater in treatments with high alkaloid concentration in *D. buzzatii* while CVs in *D. koepferae* were lower as both alkaloid or non-alkaloid fractions got more concentrated in the rearing medium. Higher performance scores were negatively associated with higher CVs indicating that a more efficient exploitation of the rearing media was related to small variation among replicates, an indication of low environmental stress [67]. These results support our predictions based on previous field and laboratory studies that alkaloids may be more harmful to *D. buzzatii* than to *D. koepferae* [45]. *D. koepferae* and *D. buzzatii* are differentially attracted to *T. terschekii* and *O. sulphurea* respectively [34], [36], [40].. Field studies have shown that *D. buzzatii* primarily uses rotting cladodes of prickly pears while *D. koepferae* uses columnar cacti [35] [40] and that *D. buzzatii* females lay more eggs on prickly pears whereas *D. koepferae* prefer to oviposit on columnar cacti [15].. Laboratory experiments also provided evidence of the pervasive and differential effect of cactus hosts on relative performance of *D. buzzatii* and *D. koepferae*. Both species are more viable, develop faster and showed increased mating success when reared in their respective primary host [34], [68], [69]. Moreover, adult flies of both species reared on the secondary host plant exhibit increased levels of wing fluctuating asymmetry [39], long considered a measure of developmental instability.

Previous studies have attributed the effects of cactus hosts to differences in chemical composition between hosts, i.e. both toxicological and nutritional properties of the environments offered by different host plants to growing larvae [35], [36], [39]. We confirmed that the presence of alkaloids is one key factor mediating the differential performance in *D. buzzatii* and *D. koepferae*. Although the metabolic pathways affected remain unclear, it is known that some alkaloids block steroid metabolism or assimilation of phytosterols [70], [71] and that alkaloid ingestion during larval life may negatively affect viability during metamorphosis [45], [72].

Before discussing the implications of our results, we would like to address the viability decline and extension of developmental time expressed by *D. koepferae* in experimental media (control, A1−A3 and NA1-NA3) when compared with overall performance in cactus media (Table 1). Similar trends were reported in previous studies [34], [41] suggesting that the nutrients required by *D. koepferae* are missing in *Drosophila* instant medium used to prepare the experimental media (potato flakes, commercial yeast, agar, glucose), at variance with our observations in *D. buzzatii.* In fact, these results are in line with our proposal that *D. koepferae* is a specialist and that *D. buzzatii* is a more generalist species. Nonetheless, these observations do not affect our conclusion of differential effects of alkaloids and the artificial cactus host (see below) on *D. buzzatii* and *D. koepferae*.

The evolution of cactophily suggests that acquisition of the capacity to degrade an array of toxic compounds present in rotting cacti could be considered an ecological apomorphy of the Neotropical lineage comprising the *repleta*, *nannoptera* and *mesophragmatica* species groups [32]. This ability allowed some subgroups to invade and diversify in cactus deserts, areas generally unfavourable for other *Drosophila* [24], [25]. Within the *repleta* group, the *D. buzzatti cluster*, which includes *D. buzzatii, D. koepferae* and at least five other Neotropical species, evolved from an ancestral *Opuntia-*feeder species that eventually began to exploit columnar cacti [32]. Therefore, *D. buzzatii* may represent the plesiomorphic state of host use compared to *D. koepferae* and all other extant species, that form the so called “serido sibling set”, which specialized in the exploitation of different columnar cacti [32], [33], [73]. However, this may not be true since ancestral character state reconstruction of host plant use in the *repleta* group indicated no phylogenetic structure [32]. In addition, though host plant use in the *fasciola* species subgroup is poorly known, some members of this basal lineage within the *D. repleta group* [32], use epiphytic species of the genus *Rhipsalis*, which is closely related to the Cactoidea [74] and other arboreal cacti as well as fruits, flowers, and fungi [24].

Alkaloids may be a determinant of patterns of host plant use in the *D. buzzatii* cluster. *D. koepferae* has evolved the ability to use a wide array of columnar cacti in the genera *Cereus, Trichocereus,* and *Neoraimondia* [75] which produce alkaloids, whereas *D. buzzatii* is more specialized on the relatively homogeneous habitat offered by prickly pears [76]. The chemical differences between cactus types may condition the direction of host shifts; a host shift would be easier for *D. koepferae* than for *D. buzzatii* since it may imply a shift from a more toxicological environment, as *T. terschekii*, to a less demanding one, as *O. sulphurea* [76].

Host shifts are fundamental components of diversification in the evolution of plant-herbivore interactions. To assess the potential of host shifts that mediate speciation it is crucial to unveil the mechanisms involved in the efficient exploitation of novel resources by specialists [19], [77], [78], especially in the critical initial phase of a recently assembled new plant– herbivore interaction. Unfortunately, host plant specialists shifting to new hosts are rarely directly observed in nature [79], [80]. Here we tested this hypothesis by creating an artificial “novel host”, an ecological opportunity in the form of a cactus, nutritionally equivalent to the prickly pear *O. sulphurea* but with the alkaloid content and concentration of the columnar *T. terschekii*. Thus, both species were exposed to a nutrient-rich medium to which *D. buzzatii* is well adapted with the addition of a toxic compound to which *D. koepferae* is more familiar. Paradoxically, and despite the observation that *D. buzzatii* was most affected by alkaloids, it performed better in the alkaloid containing artificial host. Surprisingly, *D. koepferae* suffered a dramatic reduction in performance, especially in terms of viability, and exhibited a substantial increase in the CV of performance in comparison with the other treatments or its sibling. We predicted that this host shift should have been a toxicological challenge similar to its primary host but in an *Opuntia*-like nutritional environment. These results suggest that alkaloid tolerance of *D. koepferae* may be dependent on other components of the nutritional environment.

What are these nutritional differences between cacti? For instance, the profile of fatty acids is substantially different between hosts [76], [51]. Besides, *Opuntia* species contain larger amounts of free sugars [42] than columnar cacti that have a complex chemistry that includes the presence of toxic alkaloids and other potentially toxic compounds like atypical fatty acids and triterpenes [10], [42], [51], [81]. Fermenting tissues of *O. sulphurea* and *T. terschekii* also differ in the yeast community associated to the decaying process in nature (Mongiardino Koch personal communication). Thus, one possible explanation may be that the presence of alkaloids affected a key nutritional component (a particular yeast species) rendering rotting *O. sulphurea* a nutritionally deficient medium for *D. koepferae* but not for *D. buzzatii*.

The diversification of the cactophilic *D. buzzatii* species cluster has involved a history of specialization to columnar cacti and alkaloid tolerance from a more generalist ancestral stock resembling the extant *D. buzzatii*. It remains to be determined how many independent host shifts to columnar cacti there have been and to understand the physiological mechanisms involved in specialization to reveal the evolutionary history of these flies.
